# Factors influencing student choice of a degree in physiotherapy: a population-based study in Catalonia (Spain)

**DOI:** 10.7717/peerj.10991

**Published:** 2021-04-01

**Authors:** Andrea Fuente-Vidal, Jaume-Miquel March-Amengual, Dyego L. Bezerra de Souza, Ester Busquets-Alibés, Silvia Sole, Sergi Cañete, Javier Jerez-Roig

**Affiliations:** 1 University School for Health and Sports (EUSES), Interuniversity degree in Physiotherapy, University of Barcelona and University of Girona, Spain; 2 Research group on Methodology, Methods, Models and Outcomes of Health and Social Sciences (M_3_O), Faculty of Health Sciences and Welfare, Centre for Health and Social Care Research (CESS), University of Vic-Central University of Catalonia (UVic-UCC), Vic, Barcelona, Spain; 3 Càtedra de Bioètica Fundació Grífols, UVic-UCC, Vic, Barcelona, Spain; 4 Federal University of Rio Grande do Norte, Department of Collective Health, Graduate Programme in Collective Health, Natal, Rio Grande do Norte, Brazil; 5 Faculty of Nursery and Physiotherapy, GESEC Research Group, University of Lleida, Lleida, Spain

**Keywords:** Physiotherapy, University, Gender, Origin, Career choice, Motivation, Students, Undergraduate, Physical therapy, College

## Abstract

**Background:**

In other healthcare professions, there has been extensive research into students’ motivation, but studies aiming to determine what leads individuals to choose a degree in physiotherapy are scarce. This research study had three main objectives: to obtain a sociodemographic profile of first-year physiotherapy students in Catalonia; to determine the factors that lead individuals to choose a degree in physiotherapy; and to determine potential differences, according to gender and country of origin.

**Methods:**

This is an observational, cross-sectional, multicentre study. Data were collected by means of a self-administered, *ad-hoc* questionnaire, consisting of 15 Likert scale questions, options ranging from “not influencing at all -1-” to “extremely influencing -5-”. Ten out of the twelve universities in Catalonia (Spain) that offer a degree in physiotherapy participated in this study. The sample consisted of 941 first-year physiotherapy students (55.2% men; mean age 20.1, SD: 3.4).

**Results:**

The most determinant factors leading individuals to pursue a degree in physiotherapy were: helping others (95.6%); the relationship between physiotherapy and sports (79%); physiotherapy involving manual work (76.4%); and it being perceived as providing multiple job opportunities (75.9%). Male and French students were attracted due to its relation to sports (MD = 0.369, *p* < 0.001 and MD = 0.130, *p* < 0.027), perception of it being an easy degree (MD = 0.148, *p* < 0.001 and MD = 0.091, *p* < 0.037), admiration for a known physiotherapist (MD = 0.223, *p* = 0.006 and MD = 0.265, *p* = 0.001), employability (MD = 0.297, *p* < 0.001 and MD = 0.706, *p* < 0.001), good income (MD = 0.190, *p* = 0.002 and MD = 0.609, *p* < 0.001) and social recognition (MD = 0.164, *p* = 0.011 and MD = 0.286, *p* < 0.001). Helping others (MD = −0.149, *p* < 0.001) and interest in the sciences (MD = −0.164, *p* = 0.030) were more determinant for female students. Male students were more guided by recommendation (MD = 0.234, *p* = 0.001) and to complement previous studies (MD = 0.237, *p* = 0.016). French students tended to present more interest in the selection of physiotherapy as a wish since childhood (MD = 0.595, *p* < 0.001), due to its multiple job opportunities (MD = 0.427, *p* < 0.001) and because of manual work, and did not choose it to complement previous studies (MD = −1.122, *p* < 0.001).

**Conclusions:**

The desire to help and care for others, the relation to sports, and involving manual work are the predominant factors that lead students to pursue a degree in physiotherapy. Female students favour helping others and science, whereas male students favour its relation to sports, complementing studies, social factors (admiration, recommendation, friendship) and socioeconomic determinants such as employability, good income or social recognition. When compared to Spanish students, French students were more motivated by its connection to sports, social and socioeconomic factors and some vocational determinants such as being a wish since childhood and interest in a manual profession.

## Introduction

Physiotherapy student numbers seem to be rapidly growing in Spain. Entry-level physiotherapy studies in this country currently consist of a 4-year, undergraduate university programme, comprising 240 ECTS (European Credit Transfer Accumulation System) (Asociación Española de Fisioterapeutas, 2019). This fulfils the recommendations by the World Confederation of Physical Therapy, which establishes that physiotherapy “educational programmes be based on university or university level studies, of a minimum of four years, independently validated and accredited as being at a standard that accords graduates full statutory and professional recognition” (World Confederation of Physical Therapy, 2019).

In other healthcare professions, there has been extensive research into students’ motivations, but studies aiming to determine what leads individuals to choose a degree in physiotherapy are scarce. In 2017, research was published which examined the preferences and expectations of 11,072 medicine students, 7,238 of which were in their first year (Mayta-Tristán et al., 2017). The volume of related research in medicine studies recently led Goel et al. (2018) to the publication of a systematic review. In physiotherapy, one of the few available indexed studies on the matter is that of Gotlib et al. (2011) which involved 667 students from different European countries, including Spain. This was, to the best of our knowledge, the largest multicentre study of its kind having been published on the topic. As far as we know, there are no studies comparing genders and nationalities on the field of physiotherapy undergraduate students. Understanding factors that contribute to students’ selection of a physiotherapy degree may be important to student success, higher education and industry.

The main objective of this study was to determine the factors that lead individuals to enrol in an undergraduate physiotherapy programme in Catalonia, Spain. The study also aimed to obtain a sociodemographic profile of participants, as well as to detect potential differences in motivations, based on gender and origin of the students (France *versus* Spain).

## Materials and Methods

### Design

This is an observational, multicentric, cross-sectional study, focusing on quantitative data analysis. All physiotherapy faculties in Catalonia were invited to participate. 10 (7 private; 2 public; 1 combined) accepted and 2 (1 private; 1 public) declined. The survey took place in September 2018, within the first three weeks of the students’ first academic semester. Self-administered, *ad-hoc* questionnaires were handed out in paper format. In order to accommodate for different nationalities, four language versions were offered: English, Spanish, French and Catalan. Native, bilingual physiotherapists were in charge of document supervision and translation.

Based on existing studies (Gotlib et al., 2011; Öhman, Solomon & Finch, 2002; Arrigoni et al., 2014; Malgwi, Howe & Burnaby, 2005; Peñacoba Puente et al., 2004; Crick, Perkinton & Davies, 2014; Haslach et al., 2018; Usher et al., 2013; Mooney, Glacken & O’Brien, 2008; Wouters et al., 2017; Turner, 2001; Vaartstra et al., 2017), interviews with students and expert advice, a self-reporting questionnaire was developed. The questionnaire consisted of a series of demographic data and included 15 questions, specifically designed to determine relevant factors that lead individuals to choose an undergraduate programme in physiotherapy. Students were asked about gender, age, previous university studies, origin (only France and Spain were included for bivariate analysis), reason for leaving their city if appropriate, occupation and funding sources. Using a five-point Likert scale, subjects rated the influence of 15 items, with answers ranging from “not influencing at all -1-”, to “extremely influencing -5-”. The items included: wishing to become a physiotherapist since childhood, manual work, being with a friend, admiration for a known physiotherapist, helping others, being interested in sciences, following recommendations, failing to enter their preferred course of study, searching for an easy degree, expecting good employability, good income, multiple job opportunities, social recognition, complementing previous education or work, physiotherapy’s relation to sports.

### Ethical approval

Participation in this study was voluntary. All collected information was anonymous; no identifiable data from its participants were collected. Nevertheless, the study was granted approval by two different ethics committees: Comité de Ética de la Investigación at University of Vic—Central University of Catalonia (CER-UVic-UCC) (26 June 2018; code 53/2018) and Comité de Ética y Bioseguridad de la Investigación at the University of Girona (27 July 2018; code CEBRU0010-2018). Additionally, collaboration statements were signed with all ten participating institutions and written informed consent sheets were distributed among participants.

### Eligibility criteria

Inclusion criteria included: being 18 years or older and being enrolled as first year students within a physiotherapy degree in Catalonia (Spain), during the academic year 2018–2019.

Potential participants were not considered eligible if they were unable to understand the questionnaire (available in Spanish, Catalan, English and French) or were enrolled in a double minor (e.g., combination of physiotherapy and other fields, such as nutrition or sports sciences).

### Participants and sample size

The number (universe) of physiotherapy students in Catalan universities is estimated at 1,290 individuals distributed in 12 faculties of physiotherapy (Ministerio de Educación, Cultura y Deporte, 2019). Ten of these faculties, accounting for 1,088 (84.3%) first year physiotherapy enrolments, agreed to collaborate. 45 (4.1%) students refused to participate, 99 (9.1%) were excluded for being underage (<18 years old) and 3 (0.3%) were excluded for being enrolled in a double minor. 941 filled-in questionnaires were collected from eligible candidates (*n* = 941) who voluntarily agreed to participate in the survey. The overall return rate was 86.5%, ranging from 71.0% to 92.7%. Consequently, the measure for population response rate was 941/1,290, 72.94%. A sample size of 874 individuals is necessary to detect inter-group mean differences of 0.140 points or above for a 95% confidence interval and power of 80%.

### Data analysis

Descriptive analysis was undertaken for the overall sample (*n* = 941), reporting absolute and relative frequencies for categorical variables and mean and standard deviation (SD) for quantitative variables. Data followed a normal distribution with the Kolmogorov-Smirnoff test. In order to establish any potential differences between genders and origin, data were analysed using the student’s *t*-test statistical formula. Statistical analysis was performed using the Statistical Package for the Social Sciences (SPSS) software, version 23, and *p*-values below 0.05 were considered to be significant.

## Results

The sample consisted of 519 (55.2%) men and 421 (44.8%) women. The average age was 20.7 (SD: 3.44). Student ages ranged from 18 to 49, with most individuals (81.6%) falling within the 18–21 range. 490 (52.4%) of the surveyed students came from Spain, while 425 (45.5%) came from France and 20 (2.1%) from other territories; the latter were not considered for bivariate analysis according to origin. 330 (35.1%) respondents did not have to change their habitual residence, whereas 160 (17.0%) of the respondents, despite living regularly in Spain, had to move to study at their current university. Overall, 64.3% of the total respondents were displaced from their usual residence.

For 616 (65.5%) students this was their first time at university, whereas 324 (34.4%) of them had previously undertaken some kind of university studies. 82 students (8.8%) held a previous university diploma, prior to enrolling in their current physiotherapy studies. 704 (74.9%) individuals were full-time students, while 236 (25.1%) additionally had a job. With regard to their financing sources, 563 (59.8%) students primarily received funding from their parents. 145 (15.4%) had a bank loan; 89 (9.5%) students paid for their studies themselves; and 28 (3%) were on a student grant. Sociodemographic characteristics of the participants are displayed in Table 1.

**Table 1:** Sociodemographic characteristics of participants (*n* = 941).

**Variables**	***n* or mean (% or standard deviation)**
Gender	
Males	519 (55.2)
Females	421 (44.7)
Missing	1 (0.1)
Age (years), range 18–49	20.1 (3.4)
Country of origin	
Spain	490 (52.1)
France	425 (45.2)
Other	20 (2.1)
Missing	6 (0.6)
Questionnaire language	
French	452 (48)
Spanish	316 (33.6)
Catalan	169 (18)
English	4 (0.4)
First time at university	
Yes	616 (65.5)
No	324 (34.4)
Missing	1 (0.1)
Previous university degree	
No	616 (90.5)
Yes	82 (8.7)
Missing	7 (0.7)
Occupation	
Study only	704 (74.8)
Work and study	236 (25.1)
Missing	1 (0.1)
Funding source	
Parents	563 (59.8)
Loan	145 (15.4)
Multiple	116 (12.3)
Own	89 (9.5)
Scholarship	28 (3.0)

Table 2 shows absolute and relative frequencies on students’ responses regarding how different decision factors influenced their choice of study. In absolute numbers, the most determinant factors (expressed from 1–5) for men were: helping others (4.54), relation to sports (4.28), interest in the sciences (3.48) and social recognition (3.23). The main factors for women were: helping others (4.69), manual work (4.03), multiple job opportunities (3.95) and relation to sports (3.91).

**Table 2:** Absolute and relative frequencies of students based on the different factors determining enrolment in a physiotherapy degree in Catalonia, Spain (*n* = 941).

**Determining factors**	**Not influencing at all(1)*n* (%)**	**Slightly influencing(2)*n* (%)**	**Somewhat influencing(3)*n* (%)**	**Very influencing(4)*n* (%)**	**Extremely influencing (5)*n* (%)**
Wish since childhood	195 (20.7)	139 (14.8)	251 (26.7)	236 (25.1)	119 (12.7)
Manual work	13 (1.4)	33 (3.5)	185 (18.7)	461 (49.1)	256 (27.3)
Friendship to other student	788 (83.8)	95 (10.1)	38 (4.0)	15 (1.6)	4 (0.4)
Admiration for a physiotherapist	104 (11.1)	126 (13.4)	223 (23.7)	304 (32.3)	184 (19.6)
Help others	0 (0)	4 (0.4)	38 (4.0)	279 (29.6)	619 (65.9)
Interest in the sciences	31 (4.2)	72 (9.8)	219 (29.8)	285 (38.8)	128 (17.4)
Recommendation	342 (36.3)	243 (25.8)	229 (24.3)	111 (11.8)	16 (1.7)
Not first choice	776 (82.6)	62 (6.6)	39 (4.1)	28 (3.0)	35 (3.7)
Easy degree	639 (68.0)	230 (24.5)	64 (6.8)	6 (0.6)	1 (0.1)
Employability	142 (15.2)	130 (13.9)	282 (30.2)	296 (31.7)	83 (8.9)
Good income	58 (6.2)	99 (10.6)	400 (42.7)	336 (35.9)	44 (4.7)
Multiple job opportunities	8 (0.9)	34 (3.6)	184 (19.7)	438 (46.8)	272 (29.1)
Social recognition	66 (7.1)	142 (15.2)	362 (38.7)	310 (33.1)	56 (6.0)
Complement to previous studies	417 (44.8)	90 (9.7)	129 (13.9)	185 (19.9)	110 (11.8)
Relation to sports	11 (1.2)	33 (3.5)	153 (16.3)	385 (41.1)	355 (37.9)

It can be determined that 95.6% were driven by a will to help others; 79% were influenced by physiotherapy having a strong relation to the sports world; 76.4% were strongly driven towards a degree that involved manual work; 75.9% were driven by expectations of multiple job opportunities; 56.2% stated that their decision was strongly based on their interest in the sciences; admiring a known physiotherapist was determinant for 51.9% of surveyed students; 40.6% thought that a good future income was determinant; 37.8% felt a wish to be physiotherapists since childhood; 40.6% said easy employability was a determinant factor; 39.1% said it was important that the profession is highly respected socially; 31.7% were influenced by physiotherapy being a convenient complement to their past studies/career; 13.5% chose physiotherapy because it was recommended to them; 6.7% enrolled physiotherapy even though they would have preferred to study something else and physiotherapy was not their first choice; 2% said it was important to study physiotherapy in order to accompany a friend in college; 0.7% chose physiotherapy on the grounds that it was an easy study degree.

Table 3 shows how 10 out of the 15 motivational factors that were analysed showed statistically significant differences, when analysed by gender. Men favoured the following selection factors over women: accompanying a friend (MD = 0.192, *p* < 0.001), admiring a known physiotherapist (MD = 0.223, *p* = 0.006), having been recommended to study physiotherapy (MD = 0.234, *p* = 0.001), it being an easy degree (MD = 0.148, *p* < 0.001), there being lots of employment opportunities (MD = 0.297, *p* < 0.001), obtaining social recognition (MD = 0.164, *p* = 0.011), complementing their previous studies/career (MD = 0.237, *p* = 0.016), good salary expectations, (MD = 0.190, *p* = 0.002) and its relation to sports (MD = 0.369, *p* < 0.001). In comparison to men, women were significantly more prone to choosing physiotherapy because of their interest in the sciences (MD = −0.164, *p* = 0.030) and willingness to help others (MD = −0.149, *p* < 0.001).

**Table 3:** Bivariate analysis of determinant factors in choosing a physiotherapy degree, by gender and origin.

	Gender				Nationality			
	Men mean (SD)	Women mean (SD)	Mean difference	*p* value	French mean (SD)	Spanish mean (SD)	Mean difference	*p* value
Wish since childhood	2.92 (1.30)	2.98 (1.33)	−0.059	0.493	2.27 (1.28)	1.67 (1.28)	0.595	<0.001^*^
Manual work	3.93 (0.87)	4.03 (0.83)	−0.094	0.093	3.12 (0.75)	2.83 (0.92)	0.286	<0.001^*^
Friendship with other student	1.33 (0.73)	1.14 (0.50)	0.192	<0.001^*^	0.33 (0.72)	0.18 (0.56)	0.150	0.001^*^
Admiration for a physiotherapist	3.46 (1.20)	3.24 (1.29)	0.223	0.006^*^	2.51 (1.15)	2.24 (1.30)	0.265	0.001^*^
Help others	4.54 (0.63)	4.69 (0.52)	−0.149	<0.001^*^	3.62 (0.53)	3.60 (0.64)	0.020	0.612
Interest in in the sciences	3.48 (1.06)	3.65 (0.97)	−0.164	0.030^*^	2.51 (0.94)	2.56 (1.06)	−0.056	0.474
Recommendation	2.27 (1.10)	2.04 (1.08)	0.234	0.001^*^	1.16 (1.12)	1.18 (1.07)	−0.021	0.770
Not first choice	1.34 (0.90)	1.45 (1.06)	−0.111	0.087	0.33 (0.91)	0.42 (1.01)	−0.090	0.157
Easy degree	1.47 (0.69)	1.32 (0.59)	0.148	<0.001^*^	0.45 (0.70)	0.36 (0.59)	0.091	0.037^*^
Employability	3.19 (1.17)	2.89 (1.20)	0.297	<0.001^*^	2.44 (1.18)	1.73 (1.10)	0.706	<0.001^*^
Good income	3.31 (0.88)	3.12 (0.96)	0.190	0.002^*^	2.55 (0.83)	1.94 (0.91)	0.609	<0.001^*^
Multiple job opportunities	4.03 (0.83)	3.95 (0.86)	0.074	0.180	3.23 (0.75)	2.80 (0.86)	0.427	<0.001^*^
Social recognition	3.23 (1.00)	3.07 (0.96)	0.164	0.011^*^	2.30 (0.97)	2.02 (0.99)	0.286	<0.001^*^
Complement to previous studies	2.55 (1.53)	2.31 (1.46)	0.237	0.016^*^	0.84 (1.35)	1.96 (1.42)	−1.122	<0.001^*^
Relation to sports	4.28 (0.78)	3.91 (0.94)	0.369	<0.001^*^	3.18 (0.86)	3.06 (0.89)	0.130	0.027^*^

**Notes.**

* Statistically significant (<0.05).

Eleven out of 15 factors showed statistically significant differences, according to origin. French students were significantly more inclined to choose physiotherapy due to a childhood calling (MD = 0.595, *p* < 0.001), a willingness to do a manual work (MD = 0.286, *p* < 0.001), admiration for a known physiotherapist (MD = 0.265, *p* = 0.001), good employment opportunities (MD = 0.706, *p* < 0.001), social recognition (MD = 0.286, *p* < 0.001) and relation to sports (MD = 0.130, *p* = 0.027). Students from France were also significantly more likely to wish to accompany a friend in college (MD = 0.150, *p* = 0.001) and to pursue an easy study degree (MD = 0.091, *p* = 0.037), and less inclined to choose it as a complement (MD = −1.122, *p* < 0.001), although these three factors presented low scores and small MDs.

On a 1-to-5 Likert scale, students from Spain were in general driven to study physiotherapy for the following reasons: to help others (3.60), to involve manual work (2.83), multiple job opportunities (2.80), interest in the sciences (2.56). Students from France opted for physiotherapy, primarily, for the following reasons: to help others (3.62), multiple job opportunities (3.23), relation to sports (3.18) and manual work (3.12).

## Discussion

This study managed to obtain a demographic profile of first year students of physiotherapy in Catalonia. In spite of women having historically outnumbered men in healthcare (Öhman, Solomon & Finch, 2002; Quattrin et al., 2010; Vázquez Vega, 2010; Gomá Lanzón, 2014), our study reflects a fairly balanced distribution between male and female students (55% and 45%, respectively). This could be explained by a tendency towards increasing numbers of male students in the classrooms, as previously observed by Norris et al. (2018).

According to our results, almost half of the physiotherapy students in Catalonia are foreigners; this proportion is much larger to that found in Italian classrooms (47% *vs* 12%, respectively) (Quattrin et al., 2010). Previous data suggests that as many as 1,000 French students of physiotherapy study abroad, yearly (Ordre des Masseurs Kinésitherapéutes, 2019). This could partially be explained by the difficulty students in France have in accessing a physiotherapy degree in their home country. It is likely that affordable pricing and geographical proximity make Spain an appealing destination.

In addition, approximately 65% of students had moved into the university city in order to follow their academic path and more than 1 out of 5 students already had jobs during the first few weeks of the semester. It is known that working students are more subject to stress, due to the reduced time available for studying (Oliván Blazquez, Boira Sartro & López del Hoyo, 2011), so this fact could be worth taking into consideration by educators.

The study was also able to establish how determinant given factors are in leading individuals towards a physiotherapy study programme. We now have objective data that a desire to help is what primarily moves entry-level students, followed by a perceived relation between sports and physiotherapy, using their hands and accessing a flexible work market. Helping others is a typical trait of professionals in healthcare (Quattrin et al., 2010; Vázquez Vega, 2010; Arrigoni et al., 2014). According to Camps, this is highly significant in a world where other goals -such as thriving for success, fame or wealth- are often present and can potentially harm the ultimate purpose of physiotherapy (Camps, 2007; Camps, 2015). According to Camps, the core elements to vocation include having the necessary skills (interest in experimental and human sciences, practical skills) and ethics (desire to care for others) (Camps, 2015). Our results show that these elements are robustly present in first year students. From a gender perspective, women seem to be more vocational since they presented a higher interest in the sciences and were more influenced than men by their desire to help others, which is indicative of higher altruism. Men respond more noticeably to socioeconomic motivations revolving around the workplace and economic interests, such as interest for high income or employability. In comparison to women, men are also noticeably more influenced by social determinants, such as admiration for physiotherapists, social recognition, recommendation of third parties or friendship with another student.

This data is consistent with that found by Rand et al. (2016) about social roles in the USA, as well as Rodríguez-Martínez, Sánchez-Rivas & Labajos-Manzanares (2017) on career vocation in Spain. Our findings are also in line with the reputed studies by Carol Gilligan on moral development, which showed that women have a more relational identity which makes them more prone than men to taking care of others and thus, to choose caring professions, compared to men (Gilligan, 1998). This may somehow also be related to prior findings indicating that men expect to have a higher income in the first year of employment (Johanson, 2007) and have a greater desire to own or manage a business (Öhman, Solomon & Finch, 2002; Johanson, 2007) than women.

According to our results, male students are also more interested in the relationship between physiotherapy and sports, value more studying it as a complement to previous studies and have higher expectations of it being an easy degree than female students. This desire to pursue a degree that is related to sports is also noticeably higher in French students, but they are overall less interested in studying physiotherapy as a complement to previous studies or work. The latter can be attributed to the fact that physiotherapy constitutes the first undergraduate studies for most French students.

Like men, social and economic determinants such as social recognition of the physiotherapist, expectations of high employability and income or admiration of a physiotherapist are also more noticeable among French students of both sexes. They also express a higher desire to pursue a career with “freedom” and flexible work opportunities. There are a number of aspects that could explain these cultural differences. One simple inference could be the fact that French students in Spain could already be showing a trend towards travelling and non-conventional living, thus seeking a more flexible career. But better reputation of the physiotherapy profession is also likely to be related to sociohistorical factors. Official undergraduate studies in France began in the decade of 1960s, whereas in Spain, physiotherapy did not reach the university level until 1981 (Universidad de Murcia, 2019). Moreover, the per 10,000 inhabitants ratio of physiotherapists in France is 12.6 and it is only 8.6 in Spain (Ministerio de Educación, Cultura y Deporte, 2019). Additionally, expected income in France and Spain seems to greatly differ, in favour of France and Spanish unemployment rates surpass 10% (Instituto Nacional de Estadística, 2019).

On the other hand, French students in our study showed higher scores in some factors related to vocation, since they declared a stronger vocational call from an early age and were more strongly driven towards a profession that requires manual skills. It is not surprising that approximately 38% of all students declared to have wished to be physiotherapists from an early age. It is known that individuals typically choose their career around the age of 16 (Perales, Mendoza & Sánchez, 2013). It is not age that determines the sense of vocation, but the feelings towards a given profession (Camps, 2015; Perales, Mendoza & Sánchez, 2013).

### Strengths and limitations

Limitations to this research study include the fact that the questionnaire was not validated. As far as we know, no specific validated questionnaire exists to determine factors leading individuals to pursue a degree in physiotherapy. However, the authors self-generated a questionnaire based on interviews, expert advice and previously published assessment tools. The *ad-hoc* questionnaire was translated into different languages by expert, native speakers.

There are also a number of limitations arising from the fact that determining factors were presented as a recall questionnaire (referring to deeds or thoughts of the past). Understandably, the subjectivity of participants may play a significant role, and memory bias may be present.

Regarding the representativeness of the population, it must be taken into account that a total of 99 students under the age of 18 (otherwise eligible) were not allowed to participate due to ethics requirements. Additionally, students of double minors (combined programmes of physiotherapy plus other disciplines) were also denied the opportunity to express their view. And finally, the fact that the survey was undertaken during the first days of the semester meant that late enrollers were not invited to participate.

However, the representativeness of the achieved final sample is notable. It is worth highlighting that this constitutes one of the largest studies published on factors determining the choice for a health profession and the first piece of research worldwide to thoroughly study physiotherapy selection factors, according to gender and origin.

## Conclusions

The desire to help and care for others is the predominant one in choosing to pursue a degree in physiotherapy, followed by a perceived relation between sports and physiotherapy, and the latter involving manual work. Female students are motivated more by altruism and interest in the sciences, whereas male students are motivated more by the relation to sports, physiotherapy as a complement of previous studies, social factors (admiration, recommendation, friends) and socioeconomic determinants such as employability, income or recognition. Students from France, when compared to those from Spain, were more driven by the connection with sports, social and socioeconomic determinants (expectation of good salaries, employability and multiple job opportunities, recognition and admiration) and some vocational determinants such as a vocational call from an early age and interest in a manual profession. General influencing factors as well as gender and origin differences should be further researched and acknowledged. A deeper understanding of the factors leading to a degree choice should help universities to better cater for their students and the profession.

##  Supplemental Information

10.7717/peerj.10991/supp-1 Supplemental Information 1 Raw Data

10.7717/peerj.10991/supp-2 Supplemental Information 2 Questionnaire in English language

10.7717/peerj.10991/supp-3 Supplemental Information 3 Questionnaire in French language

10.7717/peerj.10991/supp-4 Supplemental Information 4 Questionnaire in Catalan language

10.7717/peerj.10991/supp-5 Supplemental Information 5 Questionnaire in Spanish language

## Additional Information and Declarations

### Data Availability

The following information was supplied regarding data availability: Collected data are available in the Supplemental Files.

## References

[ref-1] Arrigoni C, Micheletti P, Grugnetti AM, Ferrari P, Borrelli P, Montomoli C, Pelissero G (2014). The students’ reasons to choose a nursing degree program: an Italian exploratory study. Annals of Hygienne.

[ref-2] Asociación Española de Fisioterapeutas (2019). http://www.aefi.net/Servicios/Universidades.aspx#estudios.

[ref-3] Camps V (2007). La excelencia de las profesiones sanitarias. Humanitas. Humanidades Médicas.

[ref-4] Camps V (2015). Los valores éticos de la profesión sanitaria. Education in Medicine.

[ref-5] Crick P, Perkinton L, Davies F (2014). Why do student nurses want to be nurses?. Nursing Times.

[ref-6] Gilligan C (1998). In a different voice: psychological theory and women’s development.

[ref-7] Goel S, Angeli F, Dhirar N, Singla N, Ruwaard D (2018). What motivates medical students to select medical studies: a systematic literature review. BMC Medical Education.

[ref-8] Gomá Lanzón J (2014). Imitación y experiencia.

[ref-9] Gotlib J, Białoszewski D, Opavsky J, Garrod R, Fuertes NE, Gallardo LPérez (2011). Attitudes of European physiotherapy students towards their chosen career in the context of different educational systems and legal regulations pertaining to the practice of physiotherapy: implications for university curricula. Physiotherapy.

[ref-10] Haslach SD, Aytepe Z, Kokkari A, Azrak B, Ehlers V, Herz MM (2018). Country and gender differences in the motivation of dental students—an international comparison. European Journal of Dental Education.

[ref-11] Instituto Nacional de Estadística (2019). Survey of labor insertion of university graduates 2014.

[ref-12] Johanson MA (2007). Sex differences in career expectations of physical therapist students. Physical Therapy.

[ref-13] Malgwi CA, Howe MA, Burnaby PA (2005). Influences on students’ choice of college major. Journal of Education for Business.

[ref-14] Mayta-Tristán P, Pereyra-Elías R, Montenegro-Idrogo JJ, Mejia CR, Inga-Berrospi F, Mezones-Holguín E (2017). Profile and professional expectations of medical students from 11 Latin American countries: the Red-LIRHUS project. BMC Research Notes.

[ref-15] Ministerio de Educación, Cultura y Deporte (2019). https://www.educacion.gob.es/notasdecorte/busquedaSimple.action.

[ref-16] Mooney M, Glacken M, O’Brien F (2008). Choosing nursing as a career: a qualitative study. Nurse Educ Today.

[ref-17] Norris M, Hammond JA, Williams A, Grant R, Naylor S, Rozario C (2018). Individual student characteristics and attainment in pre registration physiotherapy: a retrospective multi-site cohort study. Physiotherapy.

[ref-18] Öhman A, Solomon P, Finch E (2002). Career choice and professional preferences in a group of Canadian physiotherapy students. Advances in Physiotherapy.

[ref-19] Oliván Blazquez B, Boira Sartro S, López del Hoyo Y (2011). Estrés y otros factores psicológicos asociados en estudiantes de fisioterapia. Fisioterapia.

[ref-20] Ordre des Masseurs Kinésitherapéutes (2019). http://www.ordremk.fr/wp-content/uploads/2017/09/rapport_demographie_2017.pdf.

[ref-21] Perales A, Mendoza A, Sánchez E (2013). Vocación médica; necesidad de su estudio científico. Annals of Medicine.

[ref-22] Peñacoba Puente C, Moreno Rodríguez R, González Gutiérrez JL, Ardoy Cuadros J (2004). Perfil académico del estudiante de fisioterapia. Fisioterapia.

[ref-23] Quattrin R, Zanini A, Bulfone G, Bressan S, Farneti F, Brusaferro S (2010). Socio-demographic characteristics in healthcare students. Annals of Hygienne.

[ref-24] Rand D, Brescoll V, Everett J, Capraro V, Barcelo H (2016). Social heuristics and social roles: intuition favors altruism for women but not for men. Journal of Experimental Psychology.

[ref-25] Rodríguez-Martínez MC, Sánchez-Rivas E, Labajos-Manzanares MT (2017). Vocación ocupacional y género en estudiantes universitarios de ciencias de la salud. Revista Latinoamericana de Ciencias Sociales, Niñez y Juventud.

[ref-26] Turner P (2001). The occupational prestige of physiotherapy: perceptions of student physiotherapists in Australia. Australian Journal of Physiotherapy.

[ref-27] Universidad de Murcia (2019). https://www.um.es/historiaciencia/HistoriadelaFisioterapiaenEspanasiglosXIXyXX.pdf.

[ref-28] Usher K, West C, MacManus M, Waqa S, Stewart L, Henry R, Lindsay D (2013). Motivations to nurse. International Journal of Nursing Practice.

[ref-29] Vaartstra MB, Kercher VM, Start A, Brown AN, Peterson MD, McGrath R (2017). Understanding why undergraduate students declare and continue to study an exercise science-related major. International Journal of Exercise Science.

[ref-30] Vazquez Vega P (2010). La feminización de las profesiones sanitarias.

[ref-31] World Confederation for Physical Therapy (2019). Guideline for physical therapist professional entry level education.

[ref-32] Wouters A, Croiset G, Isik U, Kusurkar RA (2017). Motivation of Dutch high school students from various backgrounds for applying to study medicine: a qualitative study. BMJ Open.

