# Supplementary material for: Factors influencing student choice of a degree in physiotherapy: a population-based study in Catalonia (Spain)
Source: PeerJ. 2021 Apr 1;9:e10991. doi: 10.7717/peerj.10991 (PMC8019530; doi:10.7717/peerj.10991)
Supplement: Supplemental Information 2 [file peerj-09-10991-s002.pdf]

ACADEMIC AND WORK-RELATED MOTIVATIONS OF PHYSIOTHERAPY FIRST YEAR STUDENTS IN CATALONIA

Please, read the following questions and provide your answers by marking the appropriate check box with a letter X. You are kindly requested to **choose only one answer per question**, except where stated otherwise.

❖ DEMOGRAPHIC DATA.-

Are you a man or a woman? ☐ **M** ☐ **W**

Please, state your age here:

- **Have you studied at university before?** ☐ Yes ☐ No (if no, please go to the question on **Origin**)
  In which field were your university studies?☐Health care ☐Physical activity ☐Other:_________
  Did you obtain the official Degree/Diploma for your university studies? ☐ Yes ☐ No
- **Origin**
  ☐ I usually live in Spain and I did not have to move away from home, in order to study here.
  ☐ I usually live in Spain, but in order to study here I had to move out of my city.
  ☐ I usually live abroad. I moved to a different country in order to study. Specify your country: _______
- **What was the main reason why you moved away?** (If you did not move, move on to the next question)
  ☐ There are no Physiotherapy studies available in my area.
  ☐ I tried to enter Physiotherapy studies in my area, but I did not make it.
  ☐ I did not try to apply in my area. I preferred to directly move away.
  ☐ Other. Please specify ____
- **Do you work as well as study?** Please choose the option that best suits your personal situation.
  ☐ I just study.
  ☐ I study and I work.
- **Who is responsible for the payment of your study-related expenses?**
  ☐ I pay for it myself.
  ☐ My parent/s or someone in my close environment pays for it.
  ☐ I have a scholarship/grant. **Specify what type:** ☐ studies/tuition only ☐ studies and other expenses
  ☐ I asked for a bank loan, and I will have to pay the credit back later on.
- **Rate your level of Spanish language (1=minimum. 5=maximum):** ☐1 - ☐2 - ☐3 - ☐4 - ☐5
- **Rate your level of Catalan language (1=minimum. 5=maximum):** ☐1 - ☐2 - ☐3 - ☐4 - ☐5
- **Rate your level of English language (1=minimum. 5=maximum):** ☐1 - ☐2 - ☐3 - ☐4 - ☐5

❖ MOTIVATION TO BECOME A PHYSIOTHERAPIST.

Please specify TO WHAT EXTENT the following factors have influenced your decision to become a physiotherapist.

I have wanted to be a physiotherapist since I was a child.
☐Not at all ☐Slightly ☐ Somewhat ☐Very much ☐Extremely

I wish to work with my hands.
☐Not at all ☐Slightly ☐ Somewhat ☐Very much ☐Extremely

I chose Physiotherapy because a friend of mine studies it. That way we can be together.
☐Not at all ☐Slightly ☐ Somewhat ☐Very much ☐Extremely

I know some physiotherapists who I admire and I would like to be like them.
☐Not at all ☐Slightly ☐ Somewhat ☐Very much ☐Extremely

It is a profession that helps other people.
☐Not at all ☐Slightly ☐ Somewhat ☐Very much ☐Extremely

I chose it because I am interested in sciences.
☐Not at all ☐Slightly ☐ Somewhat ☐Very much ☐Extremely

I chose Physiotherapy because someone recommended that I do so.
☐Not at all ☐Slightly ☐ Somewhat ☐Very much ☐Extremely

➡

I chose Physiotherapy because I could not access my preferred course of study.
☐Not at all ☐Slightly ☐ Somewhat ☐Very much ☐Extremely

I chose Physiotherapy because I consider it to be an easy major/degree.
☐Not at all ☐Slightly ☐ Somewhat ☐Very much ☐Extremely

Unemployment rates to this profession are low.
☐Not at all ☐Slightly ☐ Somewhat ☐Very much ☐Extremely

Physiotherapists make good money.
☐Not at all ☐Slightly ☐ Somewhat ☐Very much ☐Extremely

There are many work possibilities/choices and they offer a lot of freedom.
☐Not at all ☐Slightly ☐ Somewhat ☐Very much ☐Extremely

Physiotherapists are highly respected in society.
☐Not at all ☐Slightly ☐ Somewhat ☐Very much ☐Extremely

These studies complement my previous education or job position well.
☐Not at all ☐Slightly ☐ Somewhat ☐Very much ☐Extremely

Physiotherapists are closely related to the sports world.
☐Not at all ☐Slightly ☐ Somewhat ☐Very much ☐Extremely

❖ AFTER YOUR STUDIES:

- **Do you plan on following any of these programmes? –** You may mark one or more options.

☐Another degree ☐Master's ☐Doctorate ☐Osteopathy ☐I don't know ☐Other (specify) ___________

- **In which country do you plan to work?** Pick only one answer.
  ☐ In my home country.
  ☐ In another country (please, specify) ________________________
  ☐ I don't know.
- **Which tasks would you like to carry out in the future?** Choose one or multiple answers.
  ☐ Teaching/Research.
  ☐ Management/Administration/Marketing.
  ☐ Treating patients directly (clinical practice).
  ☐ I don't know.
- **How would you like to carry out your future profession?** Choose only one answer.
  ☐ Working for my own company (self-employed).
  ☐ Working for someone else's company (employee).
  ☐ Work for my own company and also as an employee in someone else's company.
  ☐ Work for public institutions (public servant).
  ☐ I don't know.

❖ PHYSIOTHERAPY KNOWLEDGE:

Use the dotted line to write about the different HEALTH CARE BRANCHES where you think a physiotherapist can work. ……………………………………………………………………………………..…
……
……
……

Use the dotted line to write about the DIFFERENT PLACES OR TYPES OF WORK CENTRES a physiotherapist can work in. ………..………………………………………………………………………………
……
……
……

THANK YOU VERY MUCH FOR YOUR PARTICIPATION!
