# Supplementary material for: Factors influencing student choice of a degree in physiotherapy: a population-based study in Catalonia (Spain)
Source: PeerJ. 2021 Apr 1;9:e10991. doi: 10.7717/peerj.10991 (PMC8019530; doi:10.7717/peerj.10991)
Supplement: Supplemental Information 3 [file peerj-09-10991-s003.pdf]

MOTIVATIONS ACADÉMIQUES ET PROFESSIONNELLES DES ÉTUDIANTS EN PREMIÈRE ANNÉE DE KINÉSITHÉRAPIE EN CATALOGNE

S'il-te-plaît, lis attentivement les questions et réponds en cochant la case correspondante avec la lettre X.
Choisis une réponse unique pour chaque question, excepté dans les cas où le contraire est spécifié.

❖ DONNÉES DÉMOGRAPHIQUES.-

**Es-tu un homme ou une femme?** ☐H ☐F	**S'il-te-plaît, indique ici ton âge**: :

- **As-tu déjà étudié dans une université auparavant?** ☐ Oui ☐ Non (si non, passe au point **Provenance**)
  Dans quel domaine as-tu étudié? ☐Sanitaire ☐Activité physique ☐Autres ___________
  As-tu obtenu le diplôme officiel pour ces études universitaires? ☐Oui ☐Non

- **Provenance**
  ☐ Je réside habituellement en Espagne et je n'ai pas eu besoin de déménager pour les études.
  ☐ Je réside habituellement en Espagne, mais j'ai dû déménager pour venir étudier ici.
  ☐ Je réside habituellement à l'étranger. J'ai changé de pays pour faire mes études. Indique ici ton pays______

- **Quel a été le motif principal du déménagement?** (Si tu n'as pas déménagé, passe à la question suivante)
  ☐ Dans ma région, il n'y a pas d'études universitaires en kinésithérapie.
  ☐ J'ai essayé d'accéder aux études de kinésithérapie dans ma région, mais je n'ai pas réussi.
  ☐ Je n'ai pas essayé dans ma région et j'ai préféré partir directement.
  ☐ Autres. À préciser, stp____

- **Tu étudies ou tu travailles? S**'il-te-plaît, choisis l'option qui correspond à ta situation.
  ☐ J'étudie seulement.
  ☐ J'étudie et je travaille.

- **Qui finance les coûts relatifs à tes études?**
  ☐ Moi.
  ☐ Mon père/ma mère ou quelqu'un de mon entourage.
  ☐ J'ai une bourse d'études. **Indique de quel type:** ☐seuls frais d'inscription ☐frais d'inscription et autres
  ☐ J'ai demandé un crédit étudiant, dont le montant devra être remboursé plus tard.

- **Évalue ton niveau d'espagnol (1 = minimum / 5 = maximum):** ☐1 - ☐2 - ☐3 - ☐4 - ☐5
- **Évalue ton niveau de catalan (1 = minimum / 5 = maximum):** ☐1 - ☐2 - ☐3 - ☐4 - ☐5
- **Évalue ton niveau d'anglais (1 = minimum / 5 = maximum):** ☐1 - ☐2 - ☐3 - ☐4 - ☐5

❖ MOTIVATION POUR ETRE KINESITHERAPEUTE.

S'il-te-plaît, évalue COMMENT les facteurs suivants T'ONT INFLUENCÉ(E) au moment de vouloir être kinésithérapeute.

J'ai voulu être kinésithérapeute depuis mon enfance.
☐ Pas du tout ☐ Peu ☐ Moyennement ☐ Beaucoup ☐ Fortement

Je veux me consacrer à quelque chose qui implique un travail manuel.
☐ Pas du tout ☐ Peu ☐ Moyennement ☐ Beaucoup ☐ Fortement

J'ai choisi la kinésithérapie parce qu'un/e ami/e étudie la même chose, et du coup nous sommes ensemble.
☐ Pas du tout ☐ Peu ☐ Moyennement ☐ Beaucoup ☐ Fortement

Je connais des kinésithérapeutes que j'admire et j'aimerais être comme eux.
☐ Pas du tout ☐ Peu ☐ Moyennement ☐ Beaucoup ☐ Fortement

C'est une profession qui aide les autres.
☐ Pas du tout ☐ Peu ☐ Moyennement ☐ Beaucoup ☐ Fortement

Je l'ai choisie en raison de mon intérêt pour les sciences.
☐ Pas du tout ☐ Peu ☐ Moyennement ☐ Beaucoup ☐ Fortement

J'ai choisi la kinésithérapie parce qu'on me l'a recommandée.
☐ Pas du tout ☐ Peu ☐ Moyennement ☐ Beaucoup ☐ Fortement ➡

J'ai choisi la kinésithérapie parce que je n'ai pas pu accéder aux études qui me plaisaient en premier lieu.
☐ Pas du tout ☐ Peu ☐ Moyennement ☐ Beaucoup ☐ Fortement

J'ai choisi la kinésithérapie parce que les études me paraissaient faciles.
☐ Pas du tout ☐ Peu ☐ Moyennement ☐ Beaucoup ☐ Fortement

C'est une profession avec peu de chômage.
☐ Pas du tout ☐ Peu ☐ Moyennement ☐ Beaucoup ☐ Fortement

Comme kinésithérapeute on gagne bien sa vie.
☐ Pas du tout ☐ Peu ☐ Moyennement ☐ Beaucoup ☐ Fortement

Les possibilités professionnelles sont très diverses et octroient beaucoup de liberté.
☐ Pas du tout ☐ Peu ☐ Moyennement ☐ Beaucoup ☐ Fortement

Les kinésithérapeutes ont une reconnaissance sociale élevée.
☐ Pas du tout ☐ Peu ☐ Moyennement ☐ Beaucoup ☐ Fortement

Ces études complètent ma formation ou mon emploi précédents.
☐ Pas du tout ☐ Peu ☐ Moyennement ☐ Beaucoup ☐ Fortement

Les kinésithérapeutes sont étroitement liés au monde du sport.
☐ Pas du tout ☐ Peu ☐ Moyennement ☐ Beaucoup ☐ Fortement

❖ APRES LES ETUDES:

- **Tu as l'intention de suivre l'un de ces programmes? –** Tu peux choisir une ou plusieurs réponses.

☐Autre diplôme ☐Master ☐Doctorat ☐Ostéopathie ☐Je ne sais pas ☐Autres ___________________

- **Dans quel pays penses-tu travailler? –** Choisis une unique réponse.
  - ☐ Dans mon pays d'origine.
  - ☐ Dans un autre pays (à préciser) ________________________
  - ☐ Je ne sais pas.

- **Quels sont les fonctions que tu aimerais le plus développer dans ton futur professionnel? –** Choisis une ou plusieurs réponses.
  - ☐ Enseignement / Recherche.
  - ☐ Gestion / Administration / Marketing.
  - ☐ Traitement direct des patients (pratique clinique).
  - ☐ Je ne sais pas.

- **Comment aimerais-tu développer ta future profession?**– Choisis une unique réponse.
  - ☐ En travaillant à mon compte.
  - ☐ En travaillant pour le compte d'autrui.
  - ☐ A mon compte et pour autrui.
  - ☐ Comme employé de la fonction publique (fonctionnaire).
  - ☐ Je ne sais pas.

❖ CONNAISSANCES EN KINÉSITHÉRAPIE:

Écris sur les lignes pointillées LES BRANCHES DE LA SANTÉ dans lesquelles tu crois qu'un kinésithérapeute peut travailler.……………………………………………………………………………
……
……
……

Écris sur les lignes pointillées LES LIEUX OU TYPES DE CENTRE dans lesquels un kinésithérapeute peut travailler.……………………………………………………………………………
……
……
……

MERCI BEAUCOUP POUR TA PARTICIPATION!

LETTRE D’INFORMATION POUR LE PARTICIPANT

Cher/ère candidat/e:

Nous souhaitons vous inviter à participer à l'étude intitulée ***Motivations académiques et professionnelles des étudiants en première année de kinésithérapie en Catalogne.***

Je m’appelle Andrea Fuente Vidal, je suis chargée de cours universitaire et la responsable de cette étude. Ce projet est initié en coordination avec différentes universités de Catalogne et a pour objet de recueillir des informations actuelles et authentiques sur l'opinion des étudiants en kinésithérapie, leurs connaissances et leurs attentes, ainsi que les raisons pour lesquelles ils choisissent de suivre un cursus en kinésithérapie.

L’étude que je vous propose a été évaluée et validée par le Comité d'Éthique et Biosécurité de la Recherche de l'Université de Gérone, ainsi que par le Comité d'Éthique de la Recherche de l'Université de Vic - Université Centrale de Catalogne. Elle bénéficie également de l'autorisation du responsable éducatif du centre de formation dans lequel vous êtes inscrit(e).

Elle sera menée via la réalisation d'une enquête (ci-jointe). Pour participer, il est nécessaire que vous soyez majeur(e) et inscrit(e) en première année de kinésithérapie, durant l’année universitaire 2018-2019. La participation à l'enquête est totalement anonyme: il ne vous sera demandé, ni donc ne sera publié, aucune donnée d’identité personnelle. De la même manière, elle est totalement volontaire: vous avez le droit de refuser de participer et même, une fois engagé(e), de vous retirer de l’étude sans avoir à vous justifier ou exprimer quelque motif que ce soit, et ceci sans conséquence d'aucune sorte.

Si vous décidez de participer, les opinions que vous exprimerez seront traitées statistiquement. Les résultats de la recherche feront partie d'un travail de fin de Master en Activité Physique et Santé de l'Université Internationale d’Andalousie et de l'Université Pablo de Olavide. Par la suite, les résultats pourront être utilisés à fin de diffusion dans des publications rigoureusement scientifiques. Par ailleurs, si vous désirez être informé(e) des résultats, vous pouvez le solliciter par écrit à l'adresse email figurant dans mes coordonnées personnelles.

Si vous souhaitez collaborer, nous vous prions de compléter le questionnaire ci-joint et de le remettre à la personne responsable. Cette feuille d'information est pour vous.

Cordialement,

H. Fuente V.

Andrea Fuente Vidal
Investigatrice principale

Coordonnées: Andrea Fuente Vidal
N.I.F. (Numéro d'Identité en Espagne): 51079638-L
Université de Barcelone, EUSES Bellvitge
C/ Feixa Llarga, s/n
08907 Hospitalet de Llobregat. Barcelona
E-mail: cienciayfisioterapia@gmail.com
