# Supplementary material for: Factors influencing student choice of a degree in physiotherapy: a population-based study in Catalonia (Spain)
Source: PeerJ. 2021 Apr 1;9:e10991. doi: 10.7717/peerj.10991 (PMC8019530; doi:10.7717/peerj.10991)
Supplement: Supplemental Information 4 [file peerj-09-10991-s004.pdf]

MOTIVACIONS ACADÈMIQUES I LABORALS DELS ESTUDIANTS DE PRIMER CURS DE FISIOTERÀPIA A CATALUNYA

Si us plau, llegeix atentament les preguntes i respon marcant la casella corresponent amb una lletra X. **Tria una única resposta per a cada pregunta**, excepte als casos en que s'especifiqui el contrari.

❖ DADES DEMOGRÀFIQUES:

Ets home o dona? ☐ **H** ☐ **D**

Si us plau, indica la teva edat:

- **Has estudiat abans a una universitat**? ☐ Si ☐ No (si marques no, passa al punt **Procedència**)
  De quin àmbit són els teus estudis universitaris? ☐ Sanitat ☐ Activitat física ☐ Altres ___________
  Vas obtenir la titulació oficial pels teus estudis universitaris? ☐ Si ☐ No
- **Procedència**
  ☐ Solc residir a Espanya i no he tingut que canviar de casa per a estudiar.
  ☐ Solc residir a Espanya, però em vaig mudar de ciutat per a estudiar aquí.
  ☐ Solc residir a l'estranger. Vaig canviar de país per a estudiar. Indica aquí el teu país_________________
- **Quin va ser el motiu principal per mudar-te?** (Si no et vas mudar, passa a la següent pregunta)
  ☐ A la meva zona no hi ha estudis universitaris de Fisioteràpia.
  ☐ Vaig intentar accedir als estudis de Fisioteràpia a la meva zona, però no ho vaig aconseguir.
  ☐ No ho vaig intentar a la meva zona i vaig preferir sortir directament.
  ☐ Altres. Si us plau, especifica ____
- **Estudies o treballes? S**i us plau tria la opció que millor s'adeqüi a la teva persona
  ☐ Només estudio.
  ☐ Estudio i treballo.
- **Qui finança els costs relacionats amb els teus estudis?**
  ☐ Jo.
  ☐ El/la meu/meva pare/mare o algú del meu entorn.
  ☐ Tinc beca d'estudis. **Indica de quin tipus:** ☐només taxes acadèmiques ☐ taxes i altres despeses
  ☐ He demanat un crèdit, l'import del qual hauré de retornar més endavant.

- **Valora el teu nivell d'espanyol (1 és mínim. 5 màxim):** ☐ 1 - ☐ 2 - ☐ 3 - ☐ 4 - ☐ 5
- **Valora el teu nivell de català (1 és mínim. 5 màxim):** ☐ 1 - ☐ 2 - ☐ 3 - ☐ 4 - ☐ 5
- **Valora el teu nivell d'anglès (1 és mínim. 5 màxim):** ☐ 1 - ☐ 2 - ☐ 3 - ☐ 4 - ☐ 5

❖ MOTIVACIÓ PER A SER FISIOTERAPEUTA:

Si us plau valora COM T'HAN INFLUÏT els següents factors, a l'hora de voler ser fisioterapeuta.

He volgut ser fisioterapeuta des de que era petit/a.
☐ Gens ☐ Poc ☐ Quelcom ☐ Molt ☐ Totalment

Vull dedicar-me a alguna cosa que impliqui treball manual.
☐ Gens ☐ Poc ☐ Quelcom ☐ Molt ☐ Totalment

Vaig triar Fisioteràpia perquè un/a amic/amiga meu/meva estudia el mateix, i així estem junts/es.
☐ Gens ☐ Poc ☐ Quelcom ☐ Molt ☐ Totalment

Conec fisioterapeutes als quals admiro i m'agradaria ser com ells.
☐ Gens ☐ Poc ☐ Quelcom ☐ Molt ☐ Totalment

És una professió que ajuda als altres.
☐ Gens ☐ Poc ☐ Quelcom ☐ Molt ☐ Totalment

Vaig triar Fisioteràpia pel meu interès a les ciències.
☐ Gens ☐ Poc ☐ Quelcom ☐ Molt ☐ Totalment

Vaig triar Fisioteràpia perquè m'ho van recomanar.
☐ Gens ☐ Poc ☐ Quelcom ☐ Molt ☐ Totalment

➡

Vaig triar Fisioteràpia perquè no vaig poder entrar als estudis que m'hagués agradat en primer lloc.
☐ Gens ☐ Poc ☐ Quelcom ☐ Molt ☐ Totalment

Vaig triar Fisioteràpia perquè em semblen uns estudis fàcils.
☐ Gens ☐ Poc ☐ Quelcom ☐ Molt ☐ Totalment

És una professió amb poc atur.
☐ Gens ☐ Poc ☐ Quelcom ☐ Molt ☐ Totalment

Com a fisioterapeuta es guanyen uns bons diners.
☐ Gens ☐ Poc ☐ Quelcom ☐ Molt ☐ Totalment

Les possibilitats laborals són molt diverses i atorguen molta llibertat.
☐ Gens ☐ Poc ☐ Quelcom ☐ Molt ☐ Totalment

Els fisioterapeutes tenen un alt reconeixement social.
☐ Gens ☐ Poc ☐ Quelcom ☐ Molt ☐ Totalment

Aquests estudis complementen bé la meva formació o llocs de treball previs.
☐ Gens ☐ Poc ☐ Quelcom ☐ Molt ☐ Totalment

Els fisioterapeutes estan estretament vinculats amb el món de l'esport.
☐ Gens ☐ Poc ☐ Quelcom ☐ Molt ☐ Totalment

❖ DESPRÉS DELS ESTUDIS:

- **Tens intenció de cursar algun d'aquests programes? -** Pots triar una o varies respostes.

☐Un altre grau ☐Màster ☐Doctorat ☐Osteopatia ☐No ho sé ☐Altres (especificar) _______________

- **A quin país planeges treballar? –** Tria una única resposta.

  ☐ Al meu país d'origen
  ☐ A un altre país (si us plau, especifica) ________________________
  ☐ No ho sé.

- **Quines són les funcions que més t'agradaria desenvolupar en el teu futur?** Pots triar una o varies respostes

  ☐ Ensenyança/Investigació.
  ☐ Gestió/Administració/Màrqueting.
  ☐ Tractament directe de pacients (pràctica clínica).
  ☐ No ho sé.

- **Com t'agradaria exercir la teva futura professió?** –Tria una única resposta.

  ☐ Treballant a la meva pròpia empresa (compte propi).
  ☐ Treballant a l'empresa d'un altre (compte d'altre).
  ☐ Per compte d'altre i por compte propi.
  ☐ Com a empleat públic (funcionari).
  ☐ No ho sé.

❖ CONEIXEMENT DE LA FISIOTERÀPIA:

Escriu sobre la línia puntejada LES BRANQUES DE LA SALUT en les quals creus que un fisioterapeuta pot treballar...
....
....
....

Escriu sobre la línia puntejada ELS LLOCS O TIPUS DE CENTRES en els quals un fisioterapeuta pot treballar.

....
....
....
....

MOLTES GRÀCIES PER PARTICIPAR!

FULL D'INFORMACIÓ AL PARTICIPANT

Estimat/da Participant:

Amb aquest escrit volem convidar-lo a participar en la investigació titulada ***Motivacions acadèmiques i laborals dels estudiants de primer curs de Fisioteràpia a Catalunya.***

El meu nom és Andrea Fuente Vidal, soc docent universitària i la investigadora principal de l'estudi. Aquest és un projecte que es portarà a terme de manera coordinada entre diverses universitats de Catalunya. Amb ell es pretén recopilar informació actual i veraç sobre la opinió dels estudiants de Fisioteràpia, el seu coneixement i expectatives, així com les raons per les quals escullen cursar el Grau en Fisioteràpia.

La investigació que li presento ha estat avaluada i aprovada per part del Comitè d'Ètica i Bioseguretat de la Investigació de la Universitat de Girona, així com pel Comitè d'Ètica de la Investigació de la Universitat de Vic-Universitat Central de Catalunya. Així mateix, compta amb la autorització del responsable educatiu del centre on vostè està matriculat/da.

Es portarà a terme mitjançant la impartició d'una enquesta (adjunta). Per participar, es necessari que vostè sigui major d'edat i es trobi matriculat/da en primer curs del Grau en Fisioteràpia, durant el curs 2018-2019. La participació a l'enquesta és totalment anònima; no se li sol·licitarà, i per tant no es publicarà, cap dada d'identificació personal. Així mateix, és totalment voluntària; vostè té dret a rebutjar la participació, o a retirar-se de la mateixa, sense cap expressió de causa o motiu i sense conseqüències de cap tipus.

Si decideix participar, les opinions que comparteixi seran tractades de manera estadística. Els resultats de la investigació formaran part d'un treball final al Màster en Activitat Física i Salut de la Universitat Internacional d'Andalusia i la Universitat Pablo d'Olavide. Més endavant, podran servir per a la seva difusió en publicacions rigorosament científiques. A més, si vostè desitja ser informat dels resultats, pot sol·licitar-ho per escrit al e-mail que figura junt a les meves dades personals.

Per a col·laborar, li preguem que compliment i el qüestionari adjunt i ho torni a la persona responsable. Aquest full d'informació es per a vostè.

Atentament,

A. Fuente V.

Andrea Fuente Vidal
Investigadora principal

Dades de contacte: Andrea Fuente Vidal
N.I.F.: 51079638-L
Universitat de Barcelona, EUSES Bellvitge
C/ Feixa Llarga, s/n
08907 Hospitalet de Llobregat. Barcelona
E-mail: cienciayfisioterapia@gmail.com
