# Supplementary material for: Factors influencing student choice of a degree in physiotherapy: a population-based study in Catalonia (Spain)
Source: PeerJ. 2021 Apr 1;9:e10991. doi: 10.7717/peerj.10991 (PMC8019530; doi:10.7717/peerj.10991)
Supplement: Supplemental Information 5 [file peerj-09-10991-s005.pdf]

MOTIVACIONES ACADÉMICAS Y LABORALES DE LOS ESTUDIANTES DE PRIMER CURSO DE FISIOTERAPIA EN CATALUÑA

Por favor, lee atentamente las preguntas y responde marcando la casilla correspondiente con una letra X. **Elige una única respuesta para cada pregunta**, excepto en los casos en que se especifique lo contrario.

❖ DATOS DEMOGRÁFICOS.-

¿Eres hombre o mujer? ☐ **H** ☐ **M**

Por favor, indica tu edad:

- **¿Has estudiado antes en una universidad**? ☐ Sí ☐ No (si marcas no, pasa al punto **Procedencia**)
  ¿De qué ámbito son tus estudios universitarios? ☐Sanidad ☐Actividad física ☐Otros ________
  ¿Obtuviste la titulación oficial por tus estudios universitarios? ☐Sí ☐No
- **Procedencia**
  ☐ Suelo vivir en España y no he tenido que cambiar de casa para estudiar.
  ☐ Suelo residir en España, pero me mudé de ciudad para estudiar aquí.
  ☐ Suelo vivir en el extranjero y cambié de país para estudiar. Indica aquí tu país________________
- **¿Cuál fue el motivo principal para mudarte?** (Si no te mudaste, pasa a la siguiente pregunta)
  ☐ En mi zona no hay estudios universitarios de fisioterapia.
  ☐ Intenté acceder a los estudios de fisioterapia en mi zona, pero no lo conseguí.
  ☐ No lo intenté en mi zona y preferí salir directamente.
  ☐ Otros. Por favor, especifica ____
- **¿Estudias o trabajas?** Por favor elige la opción que mejor se adecúe a tu persona
  ☐ Sólo estudio.
  ☐ Estudio y trabajo.
- **¿Quién financia los costes relacionados con tus estudios?**
  ☐ Yo.
  ☐ Mi padre/madre o alguien de mi entorno.
  ☐ Tengo beca de estudios. **Indica de qué tipo:** ☐ sólo tasas académicas ☐ tasas y otros gastos
  ☐ He pedido un crédito, cuyo importe tendré que devolver más adelante.
- **Valora tu nivel de español (1 es mínimo. 5 máximo):** ☐ 1 - ☐ 2 - ☐ 3 - ☐ 4 - ☐ 5
- **Valora tu nivel de catalán (1 es mínimo. 5 máximo):** ☐ 1 - ☐ 2 - ☐ 3 - ☐ 4 - ☐ 5
- **Valora tu nivel de inglés (1 es mínimo. 5 máximo):** ☐ 1 - ☐ 2 - ☐ 3 - ☐ 4 - ☐ 5

❖ MOTIVACIÓN PARA SER FISIOTERAPEUTA.

Por favor valora CÓMO TE HAN INFLUIDO los siguientes factores, a la hora de querer ser fisioterapeuta.

He querido ser fisioterapeuta desde que era pequeño/a.
☐ Nada ☐ Poco ☐ Algo ☐ Mucho ☐ Totalmente

Quiero dedicarme a algo que implique trabajo manual.
☐ Nada ☐ Poco ☐ Algo ☐ Mucho ☐ Totalmente

He escogido Fisioterapia porque un/a amigo/a mío estudia lo mismo, y así estamos juntos/as.
☐ Nada ☐ Poco ☐ Algo ☐ Mucho ☐ Totalmente

Conozco fisioterapeutas a los que admiro y me gustaría ser como ellos.
☐ Nada ☐ Poco ☐ Algo ☐ Mucho ☐ Totalmente

Es una profesión que ayuda a los demás.
☐ Nada ☐ Poco ☐ Algo ☐ Mucho ☐ Totalmente

Lo escogí por mi interés en las ciencias.
☐ Nada ☐ Poco ☐ Algo ☐ Mucho ☐ Totalmente

Lo escogí porque me lo recomendaron.
☐ Nada ☐ Poco ☐ Algo ☐ Mucho ☐ Totalmente

➡

Escogí Fisioterapia porque no pude entrar en los estudios que hubiera querido en primer lugar.
☐ Nada ☐ Poco ☐ Algo ☐ Mucho ☐ Totalmente

Escogí Fisioterapia porque me parecen unos estudios fáciles.
☐ Nada ☐ Poco ☐ Algo ☐ Mucho ☐ Totalmente

Es una profesión con poco desempleo/paro.
☐ Nada ☐ Poco ☐ Algo ☐ Mucho ☐ Totalmente

Como fisioterapeuta se gana un buen dinero.
☐ Nada ☐ Poco ☐ Algo ☐ Mucho ☐ Totalmente

Las posibilidades laborales son muy diversas y otorgan mucha libertad.
☐ Nada ☐ Poco ☐ Algo ☐ Mucho ☐ Totalmente

Los fisioterapeutas tienen un alto reconocimiento social.
☐ Nada ☐ Poco ☐ Algo ☐ Mucho ☐ Totalmente

Estos estudios complementan bien mi formación o puesto de trabajo previos.
☐ Nada ☐ Poco ☐ Algo ☐ Mucho ☐ Totalmente

Los fisioterapeutas están estrechamente vinculados con el mundo del deporte.
☐ Nada ☐ Poco ☐ Algo ☐ Mucho ☐ Totalmente

❖ DESPUÉS DE LOS ESTUDIOS:

- **¿Tienes intención de cursar alguno de estos programas? –** Puedes elegir una o varias respuestas.

☐Otro grado ☐Máster ☐Doctorado ☐Osteopatía ☐No lo sé ☐Otros (especificar) _________

- **¿En qué país planeas trabajar?-** Elige una única respuesta.
  ☐ En mi país de origen
  ☐ En otro país (por favor, especifica) _______________________
  ☐ No lo sé.

- **¿Cuáles son las funciones que más te gustaría desarrollar en tu futuro?** – Elige una o varias.
  ☐ Enseñanza / Investigación.
  ☐ Gestión / Administración / Marketing.
  ☐ Tratamiento directo de pacientes (práctica clínica).
  ☐ No lo sé

- **¿Cómo te gustaría desempeñar tu futura profesión?** – Elige una única respuesta.
  ☐ Trabajando en mi propia empresa (cuenta propia).
  ☐ Trabajando en la empresa de otro (cuenta ajena).
  ☐ Por cuenta ajena y por cuenta propia.
  ☐ Como empleado público (funcionario).
  ☐ No lo sé.

❖ CONOCIMIENTO DE LA FISIOTERAPIA:

Escribe sobre la línea punteada LAS RAMAS DE LA SALUD en que tú crees que un fisioterapeuta puede trabajar. ……………………………………………………………………………
……………………………………………………………………………………………………
……………………………………………………………………………………………………
……………………………………………………………………………………………………

Escribe sobre la línea punteada LOS LUGARES O TIPOS DE CENTROS en que un fisioterapeuta puede trabajar. ……………………………………………………………………………
……………………………………………………………………………………………………
……………………………………………………………………………………………………
……………………………………………………………………………………………………

¡MUCHAS GRACIAS POR PARTICIPAR!

HOJA DE INFORMACIÓN AL PARTICIPANTE

Estimado/a Candidato/a:

Con este escrito queremos invitarle a participar en la investigación titulada ***Motivaciones académicas y laborales de los estudiantes de primer curso de Fisioterapia en Cataluña.***

Mi nombre es Andrea Fuente Vidal, soy docente universitaria y la investigadora principal del estudio. Este es un proyecto que se llevará a cabo de manera coordinada entre diversas universidades de Cataluña. Con él se pretende recopilar información actual y veraz sobre la opinión de los estudiantes de Fisioterapia, su conocimiento y expectativas, así como las razones por las que eligen cursar Grado en Fisioterapia.

La investigación que le presento ha sido evaluada y aprobada por parte del Comité de Ética y Bioseguridad de la Investigación de la Universidad de Girona, así como por el Comité de Ética de la Investigación de la Universidad de Vic- Universidad Central de Cataluña. Asimismo, cuenta con la autorización del responsable educativo del centro donde usted está matriculado/a.

Se llevará a cabo mediante la impartición de una encuesta (adjunta). Para participar, es necesario que usted sea mayor de edad y se encuentre matriculado/a en primer curso del Grado en Fisioterapia, durante el curso 2018-2019. La participación en la encuesta es totalmente anónima; no se le solicitará, y por tanto no se publicará, ningún dato de identificación personal. Asimismo, es totalmente voluntaria; usted tiene derecho a rechazar la participación, o a retirarse de la misma, sin expresión de causa o motivo alguno y sin consecuencias de ningún tipo.

Si decide participar, las opiniones que comparta serán tratadas de manera estadística. Los resultados de la investigación formarán parte de un trabajo final en el Máster en Actividad Física y Salud de la Universidad Internacional de Andalucía y la Universidad Pablo de Olavide. Más adelante, podrán servir para su difusión en publicaciones rigurosamente científicas. Además, si usted desea ser informado de los resultados, puede solicitarlo por escrito en el e-mail que figura junto a mis datos personales.

Para colaborar, le rogamos que cumplimente el cuestionario adjunto y lo devuelva a la persona responsable. Esta hoja de información es para usted.

Atentamente,

A. Fuente V.

Andrea Fuente Vidal
Investigadora principal

Datos de contacto: Andrea Fuente Vidal
N.I.F.: 51079638-L
Universidad de Barcelona , EUSES Bellvitge
C/ Feixa Llarga, s/n
08907 Hospitalet de Llobregat. Barcelona
E-mail: cienciayfisioterapia@gmail.com
